# Non-Targeted Metabolomics of White Rhinoceros Colostrum and Its Changes During Early Lactation by ^1^H Nuclear Magnetic Resonance Spectroscopy

**DOI:** 10.3390/metabo14110637

**Published:** 2024-11-18

**Authors:** Gernot Osthoff, Petronella Nieuwoudt

**Affiliations:** 1 Department of Microbiology and Biochemistry, University of the Free State, Bloemfontein 9300, South Africa; 2 Care for Wild Rhino Sanctuary, Private Bag X11326, Nelspruit 1200, South Africa; petronel@careforwild.co.za

**Keywords:** rhinoceros, lactation, milk, metabolite, oligosaccharide, sialyllactose

## Abstract

Background/Objectives: Dynamic changes in components from colostrum to mature milk occur in any mammal. However, the time it takes to reach the mature milk stage differs between taxa and species, as do the final concentrations of all the components. The white rhinoceros belongs to the family Perissodactyla, of which the milk and milk metabolome of the domesticated Equidae have been studied to some detail. Metabolomic information on the colostrum and milk of the Rhinocerotidae is lacking. Methods: Colostrum and milk were obtained from seven white rhinoceroses. Of note is that it was their first parturition and all followed the same diet, two factors known to affect colostrum composition and its changes during early lactation in domesticated mammals. Milk serum was prepared by the ultrafiltration of the milk samples. Untargeted ^1^N NMR spectra were processed with Topspin 3.2, calibration was carried out according to the alanine signal and the identification of signals was carried out with Chenomx and assignments in the literature. Statistical analysis of the data was carried out using MetaboAnalyst 6.0. Results: The changes in the metabolites were followed during the first 7 days of lactation as well as on day 20. The amounts of amino acids and their derivatives, organic acids and lipid metabolites decreased over lactation, while carbohydrates and their derivatives increased. The colostrum phase ended on day 2, while the transition to mature milk seemed to be complete by day 7. From day 3 to 7, galactose metabolism and tyrosine metabolism were uprated. Of interest is the presence of the oligosaccharide 3′-sialyllactose on days 3 and 4 of lactation. Conclusions: Mainly the content of carbohydrates increased over lactation, specifically lactose. The 3′-sialyllactose content peaked on days 3 and 4 of lactation. The colostrum phase ended on day 2. The mature milk stage was reached by day 7. The galactose metabolism and tyrosine metabolism were uprated after day 3 of lactation.

## 1. Introduction

The milk composition of white rhinoceroses (*Ceratotherium simum*) was reported recently [1]. The main component is lactose at 7.93%, followed by 0.93% protein, 1.76% fat, 0.40% ash and a few minor components. The milk composition does not undergo many significant changes over a lactation period of 0.5 to 18 months. Compared to the milk composition of other Perissodactyla such as the ass (*Equus asinus*) [2,3], horse (*Equus caballos*) [4,5], zebras (*Equus zebra* and *Equus burchellii*) [6], tapir (*Tapirus terrestris*) [7] and other Rhinocerotidae like the Indian rhinoceros (*Rhinoceros unicornis*) [8] and black rhinoceros (*Diceros bicornis*) [9], the milk of the Rhinocerotidae contains lower amounts of fat, higher lactose and equal protein, and therefore also the lowest gross energy.

The metabolome of human breast milk has been studied to some detail [10,11,12]. Less is known about the milk metabolome of animals, and most reports focus on domesticated animals such as the cow (*Bos taurus*) [13,14,15], goat (*Capra hircus*) [16], sheep [16], Bactrian camel (*Camelus bactrianus*) [17], and donkey [18,19,20,21]. The information on milk metabolomics finds application in dairy herd authenticity [15], animal nutrition [22] and food quality and traceability [23]. Very little information is available for other species [24,25].

To compare research reports of milk metabolomes with each other is complicated; the focus of each may differ, because targeted or non-targeted metabolites might be investigated, and the extraction methods may differ. Researchers may focus on hydrophobic metabolites, for which analysis by gas chromatography (GC) is used [12,13], while others study hydrophilic metabolites, for which liquid chromatography (LC) is preferred [16,21]. For detection in both of these chromatographic analyses, ever-more sensitive techniques are employed, such as mass spectrometry (MS), electrospray ionization (ESI)–MS/MS, triple quadrupole/linear ion trap (QTRAP) and quadrupole time-of-flight mass spectrometry (QTOF-MS). In recent years, ^1^H nuclear magnetic resonance spectroscopy (^1^H NMR) became popular as an analytical method for the characterization, detection and quantification of metabolites [14,20]. In general, NMR has several unique characteristics such as high reproducibility, an ability to identify unknown metabolites, and the ability to obtain absolute concentrations of all detected metabolites, often without any internal standards. It is also a non-destructive method, which makes it suitable to use to analyze biological fluids such as milk. These characteristics may outweigh its relatively low sensitivity and resolution [26].

Colostrum precedes the production of mature milk in the first three days postpartum or for up to a maximum of one week. It contains immunological and growth factors in high amounts, as well as bioactive compounds that protect newborns against infections and diseases. It also helps to activate the gut function and innate immune systems and induce the development of a healthy gut microbiome in the newborn [27,28]. Colostrum composition and its changes during early lactation have been reported for donkeys by making use of LC-QTOF-MS [18], ESI–QTRAP–MS/MS [21], ultra-high-performance liquid chromatography (UPLC) and ^1^H NMR [20]. Due to the improvement in the sensitivity of these detection methods, more metabolites have been detected; Li et al. [18] identified 275 metabolites, while Yang et al. [21] expanded this number to 606. In contrast, Garhwal et al. [20] positively identified only 36 metabolites with ^1^H NMR. It is, however, difficult to compare the research of these three reports, because the samples were collected at different time intervals. Nevertheless, these researchers agreed on certain changes in milk composition over lactation as well as some metabolic pathways that were up or downregulated.

We report the changes in milk metabolites during the first 7 days of lactation of the white rhinoceros. The data also contribute to the knowledge regarding the milk of the Perissodactyla.

## 2. Materials and Methods

### 2.1. Animals and Sample Collection

Milk was obtained from seven white rhinoceroses in the Care for Wild Rhino Sanctuary, Nelspruit, Mpumalanga province of South Africa. The animals were in good health and roamed on Legogote Sour Bushveld [29], which was supplemented with lucern and teff (ratio 1:2) throughout the year. For each of the animals, it was their first parturition; the rhinoceroses were Twinkle (7 years, parturition November 2022), Sibeva (7 years, parturition May 2023), River (8 years, parturition March 2022), Wyntir (8 years, parturition February 2022), Tana (9 years, parturition November 2023), Olive (9 years, parturition August 2022) and Timbi (age 10 years, parturition December 2022). The rhinoceroses were tame. Neither tranquilization nor a milk letting agent was required during milk collection. Milk was collected daily up to day 7 and then on day 20. Milk was drawn by palpation of the teats while sustained pressure was exerted on the udder. Teats were milked out, and milk from each teat was collected separately. Approximately 30–50 mL of milk per teat was obtained and was frozen at −20 °C until analysis. Milk was thawed and mixed by swirling in a water bath at 39 °C.

### 2.2. Sample Preparation of Milk for NMR Analysis

NMR analysis was carried out according to the method of Erasmus et al. [30], with a modification for extraction from milk. Milk samples were filtered with Amicon Ultra–2 mL centrifugal units with 10 kDa membrane filters (Merck, Darmstadt, Germany). Each centrifugal unit was pre-rinsed twice with 2 mL of double distilled water at 4500× *g* for 15 min in a swing-bucket centrifuge. This served to remove any trace amounts of glycerol from the membrane filter that may have interfered with NMR signals. Then, 1 mL of milk was placed in an Eppendorf tube and centrifuged at 12,000× *g* for five minutes, and 600 μL of serum was then filtered by centrifugation at 4500× *g* for 30 min in a swing-bucket centrifuge using the above-mentioned membrane filters. To 540 μL of filtered serum, 60 μL of NMR buffer solution (1.5 M potassium phosphate solution in deuterium oxide with internal standard trimethylsily1-2,2,3,3-tetradeuteropropionic acid {TSP}, pH 7.4) was added. The sample was mixed by vortex to ensure complete homogenicity and transferred to a 5 mm NMR tube for analysis.

### 2.3. ^1^H NMR Analysis

The prepared samples were subjected to NMR spectroscopy on a Bruker Avance III HD NMR spectrometer, equipped with a triple-resonance inverse (TXI) ^1^H {15N, 13C} probe head and x, y, z gradient coils at 500 MHz. The ^1^H spectra were acquired as 128 transients in 32 K data points. The spectral width was 6002 Hz, there was an acquisition time of 2.72 s and receiver gain was set to 64. The sample temperature was maintained at 300 K and the H_2_O resonance was pre-saturated by single-frequency irradiation during a relaxation delay of 4 s with a 90° excitation pulse of 8μs. Shimming of the sample was performed automatically on the deuterium signal. Fourier transformation and phase and baseline correction were carried out automatically. The software used for NMR processing was Bruker Topspin (V3.5). NMR spectral analysis, peak annotation and quantification were carried out using Bruker AMIX (V3.9.14).

### 2.4. ^1^H NMR Processing

Phase and baseline distortions of transformed spectra were corrected by using Topspin 3.2 (Bruker BioSpin, Billerica, MA, USA) and were automatically calibrated to the alanine signal at 1.48 ppm. The identification of signals was undertaken with the use of Chenomx (Edmonton, AB, Canada) or available assignments in the literature. The peaks of identified metabolites were fitted by a combination of a local baseline and Voigt functions according to the multiplicity of the NMR signal. The missing values were imputed using the k-nearest neighbor (kNN) algorithm [31] (with k = 5). These missing values were imputed with the lowest value of ethanol detected. Each metabolite was scaled to its mean value. Data were mean-centered and unit-variance scaled before multivariate analysis. Principal component analysis (PCA) and KODAMA [32] were used to visualize the metabolomic data.

### 2.5. Data Analysis

Statistical analysis was carried out using MetaboAnalyst 6.0 [33], a web server for metabolomics data analysis and interpretation, for the unsupervised principal component analysis (PCA), orthogonal partial least squares discriminant analysis (OPLS-DA), permutation tests of OPLS-DA, hierarchical cluster analysis and box and whisker plots. For univariate analyses, analysis of variance (ANOVA), Student’s *t*-test and Fisher’s least significant difference (LSD) and fold change analyses were performed. Metabolites were identified as statistically significant differential compounds for the said group comparisons if they had a *p* < 0.05 and Log2 fold change of >0.85. Heatmaps (Pearson distance calculation and ward clustering algorithm) of the significant compounds were created. The Kyoto Encyclopedia of Genes and Genomes (KEGG) pathway database [34] was used to identify biosynthesis pathways.

## 3. Results

### 3.1. Metabolite Profile

**Figure 1:**
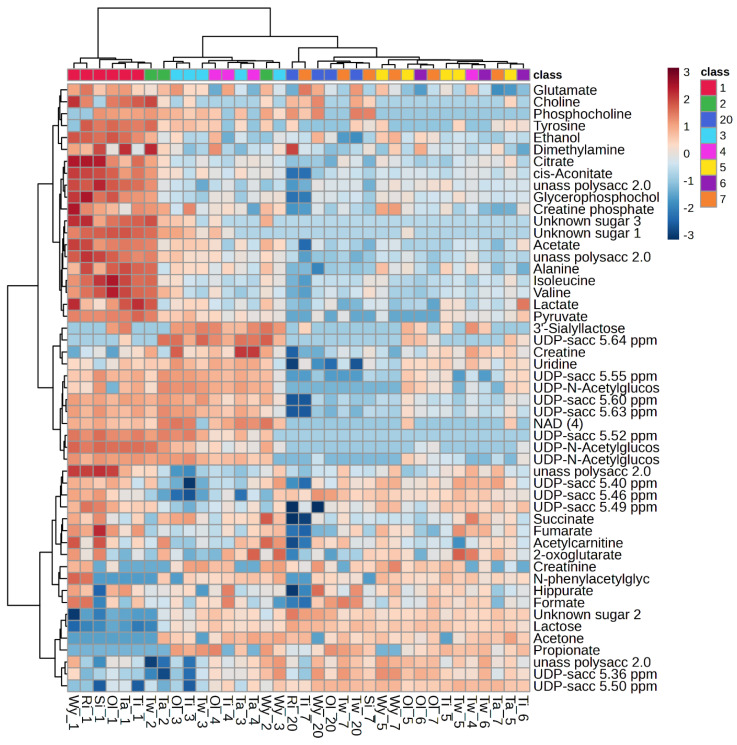
Heatmap visualization and hierarchical clustering of metabolite profiles of colostrum (day 1) and milk of white rhinoceroses on days 2 to 7 and 20 postpartum. The dendrogram represents sample clusters based on Pearson’s correlation coefficient with average linkage. Shading of blue to red indicates increasing content of the corresponding compound.

The metabolites identified in white rhinoceros milk by using Chenomx are listed in Table 1. A total of 57 metabolites were distinguished, of which 6 were unknown (Table 1). The major component of the metabolome consisted of carbohydrates, of which only lactose and 3′-sialyllactose were annotated, while the rest were only identifiable as saccharides, unknown saccharides, unknown oligosaccharides and UDP-saccharides. Furthermore, there were amino acids with derivatives, lipid derivatives, two nucleic acid derivatives, organic acids, ethanol, dimethylamine, and acetone. A heatmap (Figure 1) was used to visualize the dynamic changes in metabolite abundance in colostrum (day 1) and transitional milk samples from days 3 to 7 and 20.

All the amino acids and creatine phosphate decreased from day 1 to day 2, followed by a slower decrease thereafter. Ethanol, choline, phosphocholine and the organic acids acetate, citrate, lactate, pyruvate and cis-aconitate followed a similar trend of decreasing. Lactose, acetone and propionate increased from day 1 to day 2, followed by a slower increase thereafter. The 3′-siallyllactose increased from day 1 to day 3, followed by a decrease from day 4. The unannotated saccharides and UDP-saccharides followed any of the three trends of change.

**Figure 2:**
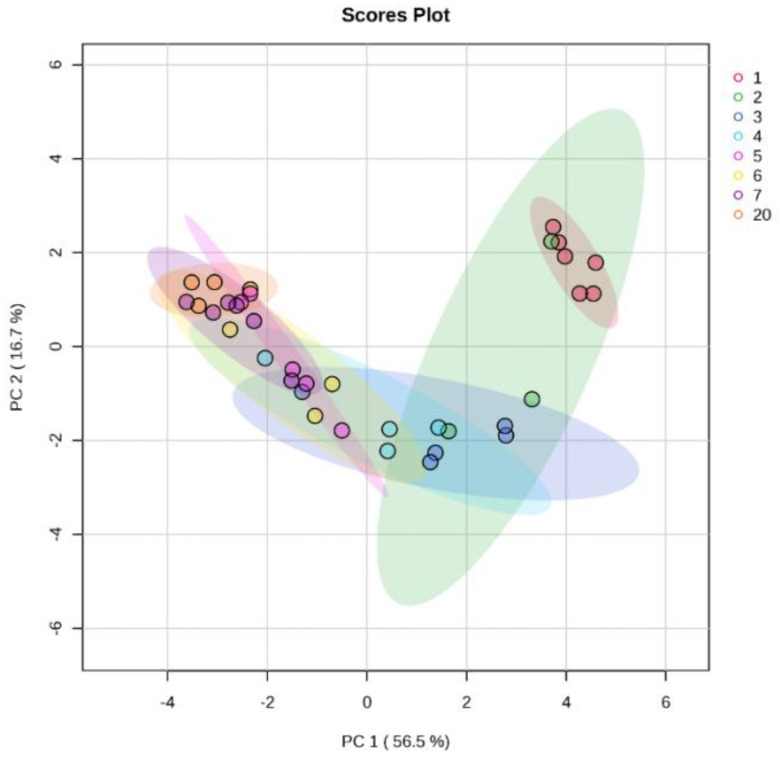
Multivariate statistical analysis of metabolites in colostrum (day 1) and transitional milk of white rhinoceroses on days 2 to 7 and 20 postpartum.

A PCA scatter plot (Figure 2) of all the white rhinoceros milk samples illustrated that the first two principal components (PC1 and PC2) accounted for 56.5% and 16.7% of the total variation, respectively. The samples were clustered within eight groups by PCA, indicating the good repeatability of the samples. There was overlap of the groups, but the PCA scores revealed a trajectory direction as lactation progressed, which confirms that lactation caused notable alterations in the milk metabolic profiles. The group of day 1 only overlapped with one sample of day 2 but was clearly separated from group 3. To a large extent, there was overlap of the groups representing days 7 and 20, while the groups of days 4–6 overlapped partially.

**Figure 3:**
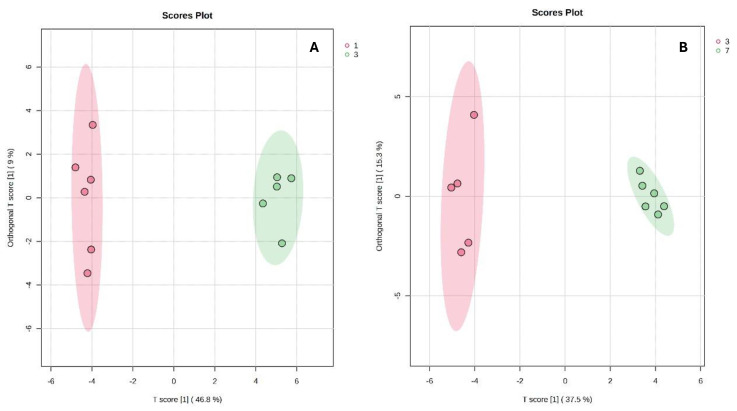
Orthogonal partial least squares discriminant analysis (OPLS-DA) score plots of the pairwise comparisons of colostrum (day 1) vs. milk from day 3 (**A**) and day 3 vs. day 7 (**B**). R2Y, Q2 and *p* values were used to validate the OPLS-DA models.

Accordingly, an OPLS-DA model was constructed to obtain a higher level of group separation. OPLS-DA score plots for pairwise comparisons of colostrum and the transitional samples were constructed and interpreted. The score plot of day 7 vs. day 20 showed that there was little difference, while the score plots of day 4 vs. day 5 and day 5 vs. day 6 showed partial differences. For our investigation, the OPLS-DA models that showed the highest values of percentage of variation (R2Y) and predictive ability (Q2) were selected, being day 1 (colostrum), day 3 and day 7 (Figure 3). The OPLS-DA models showed high-value quality parameters of R2Y *>* 0.85, Q2 *>* 0.81, and *p <* 0.05, which indicate the reliability and suitability of OPLS-DA.

### 3.2. Significantly Different Metabolites

**Figure 4:**
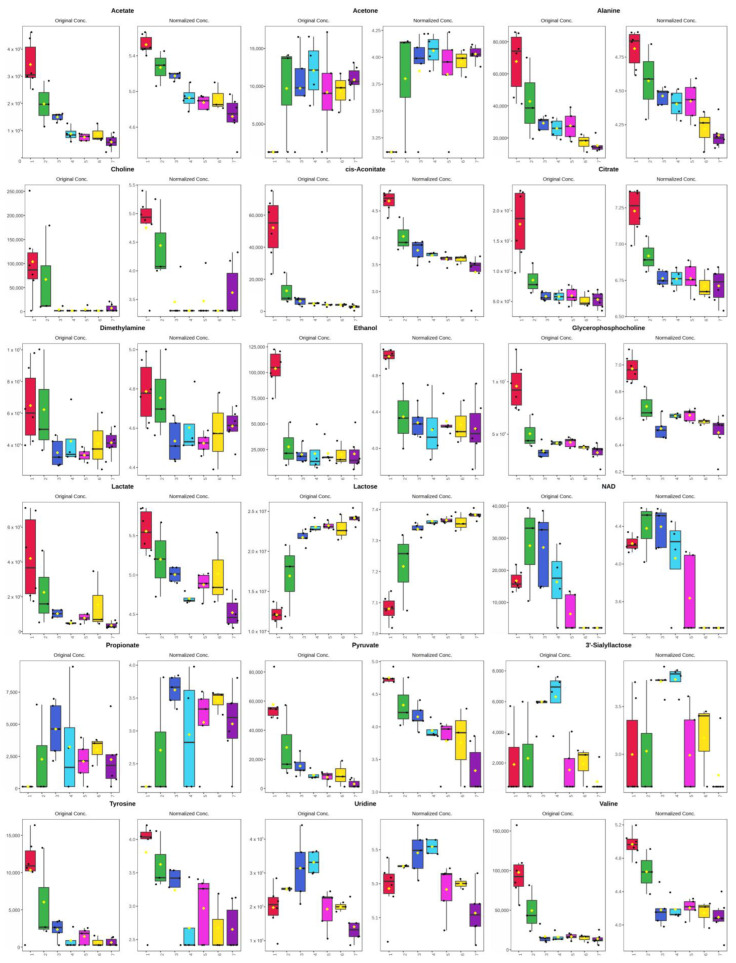
Box and whisker plots of metabolites with highest VIP scores used for KEGG enrichment. Concentrations in nM and Log nM.

VIP values and fold changes in the OPLS-DA models were used to compare the data of the colostrum (day 1) and milk samples from days 3 and 7 of the white rhinoceroses (Table 1). In total, 36 significantly different metabolites were identified. Between the groups of days 1 and 3, there were 8 upregulated and 22 downregulated, while between days 3 and 7, there were 2 upregulated and 20 downregulated. For further interpretation, the 17 unannotated saccharide metabolites had to be excluded. Of the remaining 19 metabolites, all metabolites except nicotinamide adenine dinucleotide (NAD), uridine and tyrosine were involved in the changes from day 1 to 3 and day 3 to 7 (Figure 4). The levels of NAD, uridine and tyrosine in the rhinoceros milk were high during days 1–3, but decreased thereafter. From day 1 to day 3, acetone, propionate, lactose and 3′-siallyllactose of the annotated metabolites were upregulated. None of the annotated metabolites were upregulated from day 3 to day 7.

**Figure 5:**
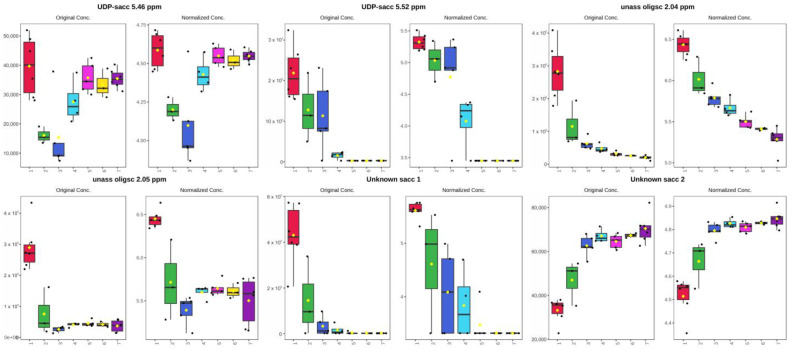
Box and whisker plots of unannotated saccharides with highest VIP scores. Concentrations in a.u. (arbitrary units) and Log a.u.

The dynamic changes in the unannotated saccharides should not go unnoticed (Figure 5). While most were downregulated, unknown saccharide 2, UDP-saccharide 5.50 ppm and UDP-N-acetylglucosamine were significantly upregulated from day 1 to day 3. UDP-saccharide 5.50 ppm and −5.46 ppm continued their upward trend from day 3 to day 7, while UDP-N-acetylglucosamine became downregulated.

### 3.3. KEGG Pathway Enrichment Analysis of Significantly Different Metabolites

**Figure 6:**
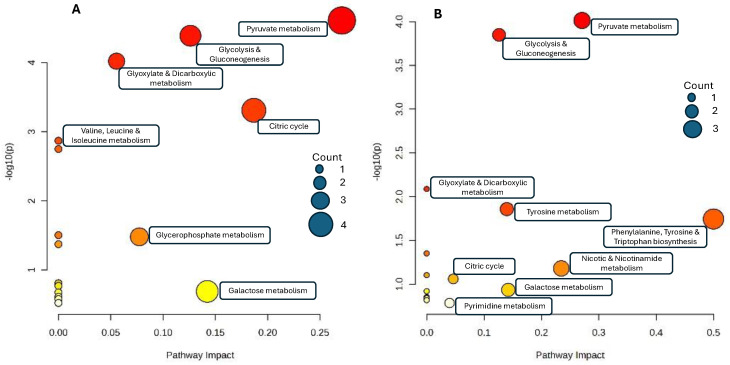
KEGG pathway enrichment analysis of different metabolites in the pairwise comparisons of colostrum (day 1) vs. milk from day 3 (**A**) and day 3 vs. day 7 (**B**).

The identified significantly different metabolites were subjected to KEGG enrichment to identify possible metabolic pathways in which they are involved. The top 16 KEGG pathways, based on *p* values, that were identified are presented in Figure 6. The most prominent pathways of day 1 vs. day 3 were pyruvate metabolism, glycolysis/gluconeogenesis, glyoxylate and dicarboxylate metabolism, citrate cycle and glycerophosphate metabolism. Of day 3 vs. day 7, the five most prominent pathways were pyruvate metabolism, glycolysis/gluconeogenesis, tyrosine metabolism, phenylalanine–tyrosine–tryptophane biosynthesis and nicotinate and nicotinamide metabolism.

### 3.4. Key Metabolites Involved in Prominent Metabolic Pathways

**Figure 7:**
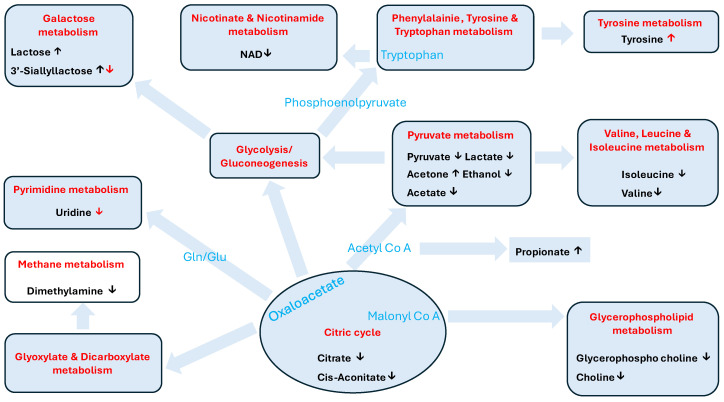
Map of metabolic pathways (red) in which the key metabolites (black) of white rhinoceros colostrum and milk are involved and which were selected by KEGG enrichment analysis. Molecules in blue represent intermediate steps. Black arrows indicate regulation between day 1 and day 3 and red arrows between days 3 and day 7. The methane metabolism pathway was not selected by KEGG enrichment.

The KEGG pathway enrichment data identified eleven metabolic pathways in which the key significantly different metabolites in white rhinoceros colostrum and milk are involved. These were pyruvate metabolism, glycolysis/gluconeogenesis, glyoxylate and dicarboxylate metabolism, citrate cycle, glycerophosphate metabolism, galactose metabolism, pyrimidine metabolism, tyrosine metabolism, phenylalanine–tyrosine–tryptophane biosynthesis, valine, leucine and isoleucine metabolism and nicotinate and nicotinamide metabolism. An overview of these metabolic pathways and the putative changes undergone by the metabolites is presented in Figure 7. The methane metabolism pathway was not selected by KEGG enrichment, although it produces dimethylamine.

## 4. Discussion

Milk from wild animals is normally obtained by opportunity, with very little chance of experimental planning. Variables that may affect the milk composition therefore have to be taken into account or avoided as much as possible. In the current case, the exact timing of milk collection is one such variable, because the animals were not always accessible at a selected postpartum time due to them hiding their calves in dense bush. This also affected the number of data points per animal. Another variable is that the suckling history before sampling was not known. Furthermore, diet and the number of parturitions may affect the milk composition of Perissodactyla [5,35]. In the current study, at least the latter two variables were avoided because all the white rhinoceroses under study roamed on the same vegetation and were fed the same supplements, and all had experienced their first parturition.

The metabolomic analysis of white rhinoceros milk resulted in the identification of 57 metabolites across seven classes, of which, 6 could not be annotated. A large number of molecules were identified as saccharides that could not be completely annotated. Nevertheless, valuable information could still be extracted from these saccharides.

The number of annotated metabolites was low compared to other metabolomic research into colostrum and milk. The ^1^H NMR-based analyses of colostrum and milk [20] described 36 metabolites from donkeys and 45 from cows [14], while as many as 270 [28] and 606 metabolytes [21] were detected in colostrum and milk from donkeys with chromatographic separation and MS detection. From these reports, it seems as if lipid-related metabolites are lacking in the ^1^H NMR-based results.

When our data on white rhinoceros colostrum and early lactation milk are compared with similar ^1^H NMR-based work on cows [14] and donkeys [20], not only did the number of detected metabolites differ, but also the composition. White rhinoceros colostrum and milk shares 18 out of 45 metabolites with cow (ethanol, alanine, isoleucine, tyrosine, valine, creatine, creatinine, lactose, acetone, acetate, fumarate, hippurate, isobutyrate, lactate, succinate, valerate, choline and glycerophosphocholine) and 13 out of 36 with donkey (alanine, isoleucine, valine, creatine, creatinine, lactose, acetate, fumarate, hippurate, isobutyrate, lactate, choline and glycerophosphocholine). In spite of this difference, the metabolites found in these two studies represent the same seven metabolite classes, as shown in Table 1. Comparison of our data with that of the chromatography and MS-based research showed only four (3′-sialyllactose, cis-aconitate, hippurate and glycerophosphocholine) [18] and four (alanine, isoleucine, valine, succinate) [21] shared metabolites. The latter two studies covered as many as 17 metabolite classes, specifically the hydrophobic ones such as coenzymes and vitamins, heterocyclic compounds, benzene and substituted derivatives and sphingolipids, to mention a few.

What complicates the comparison of studies is that colostrum was collected at varying timeframes, ranging from the first day only [14,21], the first three days [19] or the first 5 days [19,20] postpartum. The single-day collected samples showed that the assumption that the colostrum phase lasts multiple days was wrong and that valuable information was lost when samples from different days were pooled. We will therefore concentrate our discussion on research carried out on single-day sample collection.

In bovine milk [13], most metabolites, including amino acids and derivatives, organic acids and UDP-saccharides, decreased from day 1 to day 3, while glucose and lactose increased. The opposite was reported for donkey colostrum and milk [21], in which amino acids and derivatives, organic acids and saccharides increased from day 1 to day 3, while fatty acids, sphingolipids and hormones decreased. In our data, the greatest number of metabolites in the white rhinoceros milk were downregulated from day 1 to day 3 and concentrations stayed low thereafter, while only lactose increased and 3′-siallyllactose increased to peak at days 3 and 4 (Figure 4). The different changes might have a phylogenetic relationship; however, research into more species is needed to provide proof. The reduction in the large number of metabolites during the first three days of lactation might perhaps not be due to a downrated synthesis in the mammary gland cells, but to a lack of molecules that enter the alveolar lumen of the mammary gland in the paracellular way. This is brought about by the closure of the tight junctions between the mammary gland epithelial cells [36] which are composed of claudin 3 and occludin transmembrane proteins [37].

The presence of and dynamic changes in the unannotated saccharides in white rhinoceros colostrum should be noted; UDP-saccharide 5.52 ppm, unknown saccharide 1 and unassigned saccharide 2.04 ppm and −2.05 ppm decreased over the first three days of lactation, while unknown saccharide 2 increased. UDP-saccharide 5.46 ppm decreased from day 1 to day 3 and then increased from day 3 to day 7. The 3′siallyllactose, on the other hand, increased to a peak at days 3 and 4, and then decreased to zero from day 7. This is the first time that 3′-sialyllactose has been recorded in the milk of Rhinocerotidae. This oligosaccharide had been found in the milk or colostrum of many animals, including humans [38], Asian elephants [39], African elephants [40], goats [41,42], cows [43] etc. It was shown to increase from colostrum to mature milk in donkeys [22]. Changes in oligosaccharides during early lactation in breast milk, specifically the decreases, had been reported in detail for breast milk [44,45].

Some of the unassigned UDP-saccharides could be associated with galactose and lactose synthesis. Lactose is synthesized by lactose synthase, a complex of β-4-galactosyltransferase I and α-lactalbumin. This complex controls the rate of lactose synthesis [46]. Oligosaccharide synthesis, on the other hand, is catalyzed by a variety of glucosyltransferases that transfer monosaccharide residues (galactose, glucose, fructose, sialic acid and N-acetylglucosamine) to free lactose or to other saccharide residues attached to lactose [44]. In species with high levels of milk oligosaccharides, α-lactalbumin occurs at low levels [47]. Rhinoceros milk contains a high amount of lactose, which implies that the glucosyltransferases that synthesize oligosaccharides have low activity. The peaking of 3′-sialyllactose at days 3 and 4 of lactation, with a reduction thereafter, observed in our white rhinoceros milk seems to occur at the point where lactose synthesis reaches a state of high activity that promotes lactose synthesis rather than allowing any further oligosaccharide synthesis.

The metabolites in our study involved eleven metabolic pathways (Figure 7). Of these eleven, five out of fourteen were shared with breast milk, which were glyoxylate and dicarboxylate metabolism; glutathione metabolism; glycerophospholipid metabolism; and galactose metabolism [12]. Five out of nine metabolic pathways of donkey colostrum and milk were also shared with the milk and colostrum in our study: glyoxylate and dicarboxylate metabolism, citrate cycle, phenylalanine–tyrosine–tryptophane biosynthesis and valine, leucine and isoleucine metabolism [21]. Four out of ten pathways were shared with our study and another study on donkeys [18]: citrate cycle, galactose metabolism, glycerophospholipid metabolism and valine, leucine and isoleucine metabolism. Gharwal et al. [20] identified ten key metabolic pathways, of which three were also present in our rhinoceros study: pyruvate metabolism, glycolysis/gluconeogenesis and glycerophospholipid metabolism. Across these five investigations, there is very little overlap, with no single metabolic pathway being of importance in all. The greatest reason for this difference might be due to metabolites of a greater number of classes being represented in some of these studies, as was pointed out above. This resulted in a different combination of metabolic pathways being lifted out by KEGG enrichment. In general, it seems as if there is no consensus on which metabolic pathways are regulated in the progression from colostrum through intermediate milk to mature milk. Apart from the different species involved, the greatest problem is that the timeframe of milk collection differed, as was described above. The second problem which complicates any comparison is the different analytical methods that were employed. Thirdly, one may overlook the fact that the changes in metabolites during the first three days of lactation might perhaps be due to less molecules entering the alveolar lumen of the mammary gland through the paracellular way [37,38].

## 5. Conclusions

This is the first study to investigate the metabolome of colostrum and the dynamic changes in milk during the early lactation of a member of the Rhinocerotidae family. The metabolites that increased over lactation were mainly carbohydrates, specifically lactose. The oligosaccharide 3′-sialyllactose was detected on days 3 and 4 of lactation, when lactose synthesis was not uprated to full capacity. The colostrum phase was found to end on day 2; however, the transition to mature milk was only complete on day 7. After day 3 of lactation, the galactose metabolism and tyrosine metabolism were uprated. The data also provide insight into the lactation of Perissodactyla in general. To obtain a complete understanding of the metabolomes of this taxon, research on other Rhinocerotidae and Tapiridae is encouraged.

## Figures and Tables

**Table 1:** Metabolites detected in the colostrum and milk of white rhinoceroses with a fold change test (Log2 [FC]) between days 1 and 3 and days 3 and 7 along with the associated increase or decrease between the two subgroups and *t*-test for level of significance (*p* < 0.05). Open spaces indicate that the metabolites did not change significantly between the days.

	Day 1 vs. Day 3						Day 3 vs. Day 7					
Group and Metabolite	Fold Change	log2 (Fold Change)	Regulated up (↑) or down (↓)	VIP Value	t.stat	*p* Value	Fold Change	log2 (Fold Change)	Regulated up (↑) or down (↓)	VIP Value	t.stat	*p* Value
**Alcohols**												
Ethanol	0.20	−2.35	**↓**	1.37	9.75	<0.001				0.25		
**Amines**												
Dimethylamine	0.54	−0.89	**↓**	1.02	3.00	<0.01				0.63		
**Amino acid and derivatives**												
Alanine	0.43	−1.21	**↓**	1.24	5.13	<0.001				1.45	5.53	<0.001
Acetylcarnitine				0.49						0.17		
Creatine	2.15	1.11	**↑**	0.96	−2.73	<0.05				1.03		
Creatine phosphate				0.87						0.75		
Creatinine				0.51						0.44		
Glutamate				0.93						0.60		
Isoleucine	0.15	−2.75	**↓**	1.36	8.74	<0.001				0.73		
Tyrosine	0.25	−2.03	**↓**	0.59			0.26	−1.93	**↓**	1.07	3.90	<0.01
Valine	0.17	−2.55	**↓**	1.34	7.34	<0.001				0.35		
**Carbohydrate and derivatives**												
UDP-N-Acetylglucosamine (1)				0.58			0.02	−5.96	**↓**	1.24	4.66	<0.01
UDP-N-Acetylglucosamine (derivate) 1	2.07	1.05	**↑**	0.50			0.03	−4.95	**↓**	1.42	5.26	<0.001
UDP-N-Acetylglucosamine (derivate) 2	0.07	−3.94	**↓**	1.35	8.04	<0.001	0.09	−3.44	**↓**	1.37	4.87	<0.001
UDP-sacc 5.36 ppm	21.49	4.43	**↑**	0.10						0.72		
UDP-sacc 5.40 ppm				0.92	2.61	<0.05				0.76		
UDP-sacc 5.46 ppm	0.39	−1.37	**↓**	1.12	3.87	<0.01	2.31	1.21	**↑**	1.32	−3.88	<0.01
UDP-sacc 5.49 ppm	0.27	−1.88	**↓**	1.18	4.64	<0.01				1.11	−2.83	<0.05
UDP-sacc 5.50 ppm	2.16	1.11	**↑**	0.38			3.46	1.79	**↑**	1.25	−3.45	<0.01
UDP-sacc 5.52 ppm	0.53	−0.92	**↓**	0.84			0.13	−2.94	**↓**	1.32	3.96	<0.01
UDP-sacc 5.55 ppm				0.03			0.03	−5.27	**↓**	1.43	4.98	<0.001
UDP-sacc 5.60 ppm				0.55			0.15	−2.71	**↓**	1.42	5.04	<0.001
UDP-sacc 5.63 ppm				0.37			0.14	−2.81	**↓**	1.36	4.37	<0.01
UDP-sacc 5.64 ppm	21.49	4.43	**↑**	1.31	−7.14	<0.001	0.02	−5.67	**↓**		9.13	<0.001
Lactose	1.80	0.85	**↑**	1.37	−12.62	<0.001				1.37	−4.81	<0.001
3′-Sialyllactose	2.86	1.52	**↑**	1.02	−3.21	<0.05	0.13	−2.91	**↓**	1.49	7.00	<0.001
unass oligsc 2.04 ppm	0.22	−2.17	**↓**	1.36	8.60	<0.001	0.32	−1.65	**↓**	1.48	6.35	<0.001
unass oligsc 2.05 ppm	0.09	−3.48	**↓**	1.39	12.79	<0.001				0.45		
unass oligsc 2.07 ppm				0.01						0.67		
unass oligsc 2.08 ppm	0.38	−1.40	**↓**	1.21	4.93	<0.001				0.90		
Unknown sacc 1	0.08	−3.70	**↓**	1.25	4.89	<0.001	0.07	−3.76	**↓**	1.02		
Unknown sacc 2	1.89	0.92	**↑**	1.30	−6.95	<0.001				0.96		
Unknown sacc 3	0.04	−4.51	**↓**	1.28	6.05	<0.001	0.34	−1.57	**↓**	0.81		
**Carbonyls**												
Acetone	5.62	2.49	**↑**	1.17	−4.26	<0.01				0.53		
**Lipid derivatives**												
Choline	0.04	−4.61	**↓**	1.08	3.62	<0.01				0.33		
Glycerophosphocholine	0.35	−1.50	**↓**	1.33	8.04	<0.001				0.20		
N-phenylacetylglycine				0.40						0.38		
Phosphocholine				0.18						0.91		
**Nucleic acids and derivatives**												
NAD				0.76			0.12	−3.08	**↓**	1.29	11.05	<0.001
Uridine				0.86			0.48	−1.06	**↓**	1.06	4.19	<0.016
**Organic acids**												
Acetate	0.44	−1.19	**↓**	1.30	6.93	<0.001	0.39	−1.37	**↓**	1.34	4.31	<0.01
cis-Aconitate	0.12	−3.05	**↓**	1.33	7.84	<0.001	0.44	−1.17	**↓**	0.98		
Citrate	0.30	−1.72	**↓**	1.35	9.73	<0.001				0.48		
Formate				0.32						0.13		
Fumarate				1.09	3.57	<0.01				0.53		
Hippurate				0.39						0.04		
Lactate	0.25	−2.03	**↓**	1.16	4.30	<0.01	0.35	−1.51	**↓**	1.45	5.10	<0.001
2-oxoglutarate				0.11						0.11		
Succinate				0.25						0.96		
Propionate	10.76	3.43	**↑**	1.35	−11.13	<0.001	0.49	−1.04	**↓**	0.81		
Pyruvate	0.27	−1.91	**↓**	1.31	7.04	<0.001	0.20	−2.34	**↓**	1.33	4.39	<0.01
**Unassigned signal**												
6.20 ppm												
7.94 ppm												
7.95 ppm												
8.05 ppm												
8.11 ppm												
8.62 ppm												

## Data Availability

Raw data were generated at the Centre for Human Metabolomics, North-West University, South Africa. Derived data supporting the findings of this study are available from the corresponding author on reasonable request.
